# Study protocol: Insight 46 – a neuroscience sub-study of the MRC National Survey of Health and Development

**DOI:** 10.1186/s12883-017-0846-x

**Published:** 2017-04-18

**Authors:** Christopher A. Lane, Thomas D. Parker, Dave M. Cash, Kirsty Macpherson, Elizabeth Donnachie, Heidi Murray-Smith, Anna Barnes, Suzie Barker, Daniel G. Beasley, Jose Bras, David Brown, Ninon Burgos, Michelle Byford, M. Jorge Cardoso, Ana Carvalho, Jessica Collins, Enrico De Vita, John C. Dickson, Norah Epie, Miklos Espak, Susie M. D. Henley, Chandrashekar Hoskote, Michael Hutel, Jana Klimova, Ian B. Malone, Pawel Markiewicz, Andrew Melbourne, Marc Modat, Anette Schrag, Sachit Shah, Nikhil Sharma, Carole H. Sudre, David L. Thomas, Andrew Wong, Hui Zhang, John Hardy, Henrik Zetterberg, Sebastien Ourselin, Sebastian J. Crutch, Diana Kuh, Marcus Richards, Nick C. Fox, Jonathan M. Schott

**Affiliations:** 1 Dementia Research Centre, Institute of Neurology, University College London, London, UK; 2 Translational Imaging Group, Centre for Medical Image Computing, University College London, London, UK; 3 Leonard Wolfson Experimental Neurology Centre, Institute of Neurology, University College London, London, UK; 4 Institute of Nuclear Medicine, University College London Hospitals, London, UK; 5 Department of Molecular Neuroscience, Institute of Neurology, University College London, London, UK; 6 Department of Medical Sciences and Institute of Biomedicine - iBiMED, University of Aveiro, Aveiro, Portugal; 7 MRC Unit for Lifelong Health and Ageing at UCL, London, UK; 8 Lysholm Department of Neuroradiology, The National Hospital for Neurology and Neurosurgery, Queen Square, London, UK; 9 Neuroradiological Academic Unit, Department of Brain Repair and Rehabilitation, Institute of Neurology, University College London, London, UK; 10 Department of Clinical Neuroscience, Institute of Neurology, University College London, London, UK; 11 National Hospital for Neurology and Neurosurgery, London, UK; 12 Department of Computer Science and Centre for Medical Image Computing, University College London, London, UK; 13 Reta Lila Weston Research Laboratories, Department of Molecular Neuroscience, Institute of Neurology, University College London, London, UK; 14 Department of Psychiatry and Neurochemistry, Institute of Neuroscience and Physiology, the Sahlgrenska Academy at the University of Gothenburg, Mölndal, Sweden; 15 Clinical Neurochemistry Laboratory, Sahlgrenska University Hospital, Mölndal, Sweden

**Keywords:** Epidemiology, Life course, Genetics, Alzheimer’s Disease, Ageing, Magnetic resonance imaging, Positron emission tomography, Cognition, Vascular disease, Birth cohort

## Abstract

**Background:**

Increasing age is the biggest risk factor for dementia, of which Alzheimer’s disease is the commonest cause. The pathological changes underpinning Alzheimer’s disease are thought to develop at least a decade prior to the onset of symptoms. Molecular positron emission tomography and multi-modal magnetic resonance imaging allow key pathological processes underpinning cognitive impairment – including β-amyloid depostion, vascular disease, network breakdown and atrophy – to be assessed repeatedly and non-invasively. This enables potential determinants of dementia to be delineated earlier, and therefore opens a pre-symptomatic window where intervention may prevent the onset of cognitive symptoms.

**Methods/design:**

This paper outlines the clinical, cognitive and imaging protocol of “Insight 46”, a neuroscience sub-study of the MRC National Survey of Health and Development. This is one of the oldest British birth cohort studies and has followed 5362 individuals since their birth in England, Scotland and Wales during one week in March 1946. These individuals have been tracked in 24 waves of data collection incorporating a wide range of health and functional measures, including repeat measures of cognitive function. Now aged 71 years, a small fraction have overt dementia, but estimates suggest that ~1/3 of individuals in this age group may be in the preclinical stages of Alzheimer’s disease. Insight 46 is recruiting 500 study members selected at random from those who attended a clinical visit at 60–64 years and on whom relevant lifecourse data are available. We describe the sub-study design and protocol which involves a prospective two time-point (0, 24 month) data collection covering clinical, neuropsychological, β-amyloid positron emission tomography and magnetic resonance imaging, biomarker and genetic information. Data collection started in 2015 (age 69) and aims to be completed in 2019 (age 73).

**Discussion:**

Through the integration of data on the socioeconomic environment and on physical, psychological and cognitive function from 0 to 69 years, coupled with genetics, structural and molecular imaging, and intensive cognitive and neurological phenotyping, Insight 46 aims to identify lifetime factors which influence brain health and cognitive ageing, with particular focus on Alzheimer’s disease and cerebrovascular disease. This will provide an evidence base for the rational design of disease-modifying trials.

## Background

Dementia is the leading cause of death in England and Wales, accounting for 11.6% of all deaths registered in 2015 [1]. As the population ages, the burden of neurological diseases and dementia in particular will increase dramatically. Current estimates suggest that 44 million people worldwide are currently living with dementia, and this number is predicted to more than triple by 2050, by which time the annual cost of dementia in the US alone may exceed US$604 billion [2]. Estimates suggest that a five-year delay in symptom onset would halve prevalence, costs and burden [3]. Understanding the causes of dementia, and lifestyle or pharmacological interventions that can prevent or delay the onset of symptoms is therefore a global priority.

Dementia is a clinical syndrome due to many underlying diseases, of which Alzheimer’s disease (AD) is the commonest single cause. AD is characterised histopathologically by the accumulation of senile plaques mainly composed of amyloid β (Aβ), neurofibrillary tangles composed of hyperphosphorylated tau [4], and excess neuronal cell loss (atrophy) in vulnerable regions, notably the medial temporal lobe and parietal association cortices. The emergence of techniques for studying biomarkers allows for many aspects of AD pathology to be assessed in vivo. In particular, positron emission tomography (PET) using amyloid-specific tracers allows for quantification of fibrillar amyloid burden; and modern multi-modal magnetic resonance imaging (MRI) offers a non-invasive way of determining brain volumes, cerebrovascular disease, white matter tract integrity, brain perfusion, functional connectivity, and brain microstructure. Applying many of these techniques to cohorts with rare, autosomal dominantly inherited forms of AD, sporadic AD, and healthy older controls suggests that: (1) accumulation of Aβ is seen in a significant proportion (up to a third) of individuals in their 70s; (2) Aβ accumulation occurs prior to, and is likely to trigger, the development of other pathological processes core to AD, including the deposition and spread of abnormally hyperphosphorlayed tau through vulnerable networks, microglial activation, brain hypometabolism, and increased rates of atrophy; and (3) these processes all occur several years – and in the case of amyloid deposition perhaps a decade or more – prior to the development of symptoms [5, 6].

These findings have already led to important advances, including (1) a re-conceptualisation of AD to include healthy individuals at risk, with contemporary research criteria now determining that asymptomatic individuals with evidence for brain amyloid, brain amyloid + neurodegeneration, or brain amyloid + neurodegeneration + subtle cognitive impairment can be designated as having preclinical AD [7–9]; and (2) the advent of clinical trials in asymptomatic participants either at risk of AD on the basis of carrying a gene known to cause familial AD [10, 11] or individuals with asymptomatic amyloidosis [12]. These trials aim to modify aspects of AD pathology by slowing or even reversing the development of brain pathology, and delaying the onset of cognitive decline and ultimately the clinical manifestation of AD dementia.

Our understanding of this presymptomatic period is, however, far from complete. Evidence to date comes largely from extrapolation of cross-sectional (or short-interval longitudinal follow-up) studies to infer the sequence of changes that occur over much longer periods [13]. Individuals selected for such studies often are not typical of the population as a whole, with many having genetic risks factors, concerns about cognition, or a family history of dementia [14]. Little is known in the general population about the factors that influence the development, sequence and timing of the different pathologies implicated in AD, and how they interact with other brain pathologies (e.g. cerebrovascular disease) to influence cognitive function. Consequently, the evidence base to inform the design of clinical trials in the presymptomatic phase is currently limited.

In parallel with the need to identify preclinical AD for interventional trials, it is also vital to understand what influences an individual’s risk of developing AD and other forms of late-life cognitive impairment. There are more than 20 identified genetic risk factors for AD, most of which exert only a small influence on risk, but together, by way of a polygenic risk score, have been shown to almost double case prediction from chance [15]. There is evidence that education and physical exercise are protective, whereas mid-life hypertension, obesity and diabetes adversely influence risk [16]. However it is unclear whether these factors act independently, cumulatively or interactively, and how they influence different pathological processes that can lead to dementia; to address these questions requires prospective data across the life course.

The Medical Research Council (MRC) National Survey of Health and Development (NSHD) has followed 5362 individuals since their birth in England, Scotland and Wales during one week in March 1946 [17–19]. Repeated waves of data collection since childhood have enabled detailed cognitive and physical phenotyping of this population-representative cohort. Details of the cohort are provided elsewhere [17, 18], with an overview of relevant information collected to date in Table 1. Now aged 71, members of this intensively-studied cohort are at a critical age to investigate preclinical AD: old enough to be at high risk for amyloid pathology, but several years before the expected exponential rise in dementia prevalence [20].

**Table 1:** Overview of life course data available for MRC NSHD study participants

			Time point (ages)						
			1946 (birth)	1947–50 (1–4 yrs)	1951–61 (5–15 yrs)	1962–77 (16–31 yrs)	1978–2003 (32–57 yrs)	2006–10 (60–64 yrs)	2014–15 (68–69 yrs)
Number of data collections			1	2	8	8	3	1	1
Measure	Social factors	Socioeconomic position	✓	✓	✓	✓	✓	✓	✓
		Social function (contacts, support, participation)	-	-	-	-	✓	✓	✓
		Occupation	-	-	-	✓	✓	✓	✓
		Educational Qualifications			✓	✓			
	Psychological measures	Behaviour and mental health	-	✓	✓	✓	✓	✓	✓
	Physical and health measures	Survival and morbidity	✓	✓	✓	✓	✓	✓	✓
		Anthropometric measures	✓	✓	✓	✓	✓	✓	✓
		Smoking status	-	-	-	✓	✓	✓	✓
		Exercise and physical health	-	-	✓	-	✓	✓	✓
		Diet	-	✓	-	-	✓	✓	✓
		Respiratory function	-	-	-	-	✓(36, 43, 53)	✓	✓
		Cardiovascular function	-	-	-	-	✓(36, 43, 53)	✓	✓
		Musculoskeletal measures	-	-	-	-	✓ (53)	✓	✓
		Blood sample	-	-	-	-	✓ (53)	✓	✓
		Urine sample	-	-	-	-	-	✓	-
	Cognition	Cognitive function (verbal/non-verbal)	-	-	✓ (8, 11, 15)	✓ (26)	✓ (43, 53)	✓	✓

We describe here the study design and protocol of “Insight 46”, a prospective longitudinal two time-point (0, 24 month) sub-study of 500 study members, incorporating the collection of new clinical, neuropsychological, MRI, PET amyloid imaging, and blood and urine biomarkers. We outline the study’s organisation and funding structure, provide an overview of the recruitment criteria, the cognitive, imaging and fluid biomarker protocols, and the duty of care protocol. We summarise the key hypotheses to be tested, and the data that are being collected; these data will in due course be made available to the research community.

## Methods/design

### Study organisation/funding

Insight 46 is funded by grants from Alzheimer’s Research UK (ARUK-PG2014–1946, ARUK-PG2017-1946 PIs Schott, Fox, Richards), the Medical Research Council Dementias Platform UK (CSUB19166 PIs Schott, Fox, Richards), the Wolfson Foundation (PR/ylr/18575 PIs Fox, Schott), the Medical Research Council (MC_UU_12019/1 PI Kuh and MC_UU_12019/3 PI Richards), the Wellcome Trust (Clinical Research Fellowship 200109/Z/15/Z Parker) and Brain Research Trust (UCC14191, PI Schott). AVID Radiopharmaceuticals (a wholly owned subsidiary of Eli Lilly) provide the PET amyloid tracer (Florbetapir) but had no part in the design of the study.

Separate ethical approvals for NSHD have been provided by Research Ethics Committees in England and Scotland outlined elsewhere [17, 19, 21]. Ethical approval for the neuroscience sub-study was granted by the National Research Ethics Service (NRES) Committee London (REC reference 14/LO/1173, PI Schott). All participants provide written informed consent to participate and for their data to be stored in accordance with the Data Protection Act.

### Participants’ recruitment and clinical protocol description

#### Participants

To capitalise on the life course data and to avoid a priori decisions as to who might be at risk of cognitive decline, entry criteria to the sub-study are based only on maximising the life course data available for analysis. A sample of 500 NSHD study members are being selected at random from those who attended a clinic-based assessment age 60–64, had previously intimated they were willing to attend a clinic visit in London and for whom relevant data in childhood and adulthood are available. These relevant data are shown in Table 2.

**Table 2:** Minimum life course dataset for Insight 46

Attendance at a clinic visit at age 60–64
Parental socioeconomic position: at least one indicator of occupational social class or education
Cognition: memory and processing speed from the 60–64 year collection AND at least one set of measures at either ages 8, 11 or 15
Early physical growth trajectories: birth weight and at least one measure of height and weight at ages 4–15
Educational attainment: highest qualification by age 26
Mental health: teacher ratings of behaviour and temperament at ages 13 or 15, and at least one measure of affective symptoms at ages 36, 43, 53 or 60–64
Blood pressure, lung function, adult height and weight: at least one measure of each at ages 36, 43, 53 or 60–64
Health behaviours: at least one measure of smoking and physical exercise at ages 36, 43, 53 or 60–64
Blood: either age 53 or 60–64 samples

**Fig. 1:**
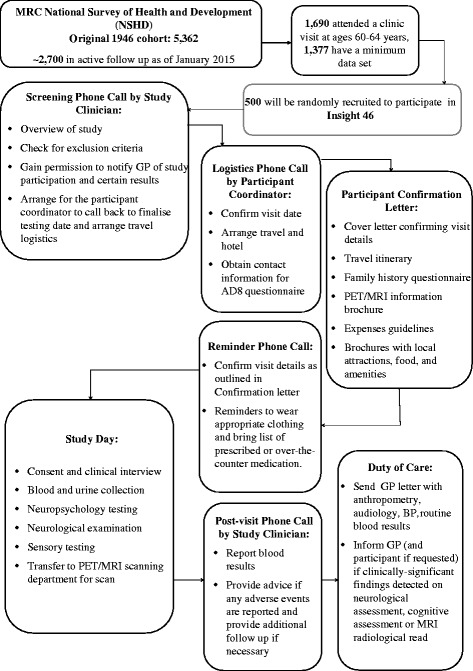
Flowchart for Insight 46

The first 500 study members fulfilling these criteria and agreeing to participate will be included. Excluded are individuals with contraindications to MRI or PET including, but not limited to, claustrophobia, metallic implants such as pacemakers, or research nuclear medicine scans within the last year that would result in an individual exceeding acceptable mandated yearly radiation exposures. Where appropriate, the option to consent to post-mortem brain donation is discussed with participants. A flowchart outlining the study is shown in Fig. 1.

#### Duty of care

A duty of care protocol building on the NSHD protocol used in 2006–10 and in accordance with the MRC/Wellcome Trust guidelines is being implemented for the purpose of feeding back health-related findings in research [22] to each participant and their GP. Participants are given the option of ‘opting out’ from receiving any correspondence regarding reportable findings, but must consent to their GP receiving the information. Anthropomorphic measures (height and weight), recumbent blood pressure, audiometry and a range of standard clinical blood tests (haemoglobin, platelet count, vitamin B12, urea, creatinine, random glucose and TSH) together with their normal ranges are routinely reported. Participants with results outside the normal range are advised to discuss the results with their GP in a timely fashion. If blood results are significantly outside the normal range, falling beyond pre-specified ‘Action’ levels, the study clinician contacts the participant and GP via telephone within 48 h of receipt of results.

All T1, T2 and FLAIR volumetric MRI sequences are reviewed by a consultant neuroradiologist at the National Hospital for Neurology and Neurosurgery. The study follows guidelines based on the UK Biobank imaging study (www.ukbiobank.ac.uk/wp-content/uploads/2016/11/Incidental-findings-list-of-possible-abnormalities.pdf), and information is only fed back to study members and their GP if there is an MRI abnormality that might require treatment or surveillance. A list of potential findings considered reportable are summarised in Table 3. The ethical challenges of providing information regarding amyloid PET results in cognitively-normal individuals has been discussed elsewhere [23], and for this study amyloid PET status is not fed back to study members.

**Table 3:** Reportable MRI brain findings

Acute brain infarction
Acute brain haemorrhage (note: not old bleeds)
Intracranial mass lesions (note: not meningiomas in locations considered highly unlikely to cause problems)
Suspected intracranial aneurysm or vascular malformation (inc. cavernomata) (note: not aneurysms less than 7 mm in diameter)
Colloid cyst of the 3rd ventricle
Acute hydrocephalus
Significant sinus disease with suspicion of underlying pathology (e.g. unilateral sinus opacification)
Other unexpected, serious, or life-threatening findings

In addition, information is fed back to participants and their GP if clinical assessments reveal clear evidence of significant cognitive impairment (based on a MMSE score ≤ 24 and/or significant concern from study clinician) or clinically detectable parkinsonism (i.e. fulfilling Queen Square Brain Bank criteria [24] for Parkinson’s disease (PD)) in previously undiagnosed individuals. Appendix 1 summarises reportable findings and normal ranges employed in Insight 46.

### Clinical, neurological, cognitive and sensory assessments

All individuals are assessed at a single site (UCL). Participants complete four self-administered questionnaires; undergo a structured clinical interview with a neurologist; have a structured neurological examination; undergo neuropsychological testing, and assessment and testing of auditory, olfactory and visual function. These assessments have been designed to be administered in divided sessions that last less than four hours during a single day (mean duration =199 min based on six pilot visits).

#### Self-administered questionnaires

*The state and trait anxiety inventory* [25]: This 40-item questionnaire assesses an individual’s thoughts and feelings, and is designed to quantify anxiety levels at the present moment and in general.

*A dental health questionnaire* [26]: There is growing evidence that periodontitis is a risk factor for sporadic AD and it has been postulated that periodontal pathogens may drive chronic neuro-inflammation contributing to Alzheimer’s pathology [27]. This self-administered questionnaire utilises eight questions designed to assess the likelihood of periodontitis.

*A handedness questionnaire* [28]: Hand preference is closely related to cerebral dominance [29]. This questionnaire asseses hand preference for 12 different tasks using a numerical scale enabling quantification of each participant’s handedness (range − 24 to +24).

*Screening question for Rapid Eye Movement (REM) sleep behaviour disorder* [30]: REM sleep behaviour disorder is a known risk factor for the emergence of Parkinson’s disease and related disorders. A yes/no answer is obtained to the question “Have you ever been told, or suspected yourself, that you seem to ‘act out your dreams’ while asleep (for example, punching, flailing your arms in the air, making running movements, etc.)?”. This has a sensitivity of 93.8% and a specificity of 87.2% for detecting REM sleep behaviour disorder [30].

#### Clinical interview

A standard personal and family history of neurological illness or cognitive impairment and a medication history is obtained. In addition, participants are screened for measures of self-perceived cognitive decline using the SCD-Q part I (MyCog) questionnaire [31] and are asked questions that enable coding of essential features of subjective cognitive decline as outlined by the working group of the Subjective Cognitive Decline Initiative [32]. A corroborative history regarding each participant’s cognitive functioning is obtained using the AD8 screening tool, an informant questionnaire administered in person or via the telephone by the study clinician. The AD8 correlates well with the clinical dementia rating scale (CDR), and has high sensitivity and specificity for detecting cognitive impairment [33, 34].

#### Physical and neurological examination

A physical examination comprises anthropomorphic measures (weight in kilograms and height measured to the nearest mm), and lying and standing blood pressure at three minutes to assess for evidence of orthostatic hypotension (OMRON HEM-905; OMRON Healthcare UK Ltd., Milton Keynes).

Patients with AD and other forms of dementia have more marked decline in motor function, including gait, than healthy controls, with the possibility that these changes may precede the onset of frank cognitive symptoms [35, 36]. This is perhaps not surprising if gait is viewed as a complex cognitive task, requiring an interplay of attention, executive function and visuospatial function, in addition to the motor processing functions of the motor cortex, basal ganglia and cerebellum. It has therefore been suggested that changes in gait and motor skills in general may reflect and correlate with early cognitive change [37].

Participants’ self-paced gait is assessed over a 20-m distance in isolation and while performing a cognitive task (single-letter-cued (phonemic) fluency and dual-letter-cued (phonemic) alternate fluency), and wearing an accelerometer on the lower back (LPMS-B inertial measurement unit (Life performance Research Inc)), with data analysis using a custom program written in LabVIEW2010 (National Instruments, Ireland). Temporal (step time and cadence) as well as spatial (step-, stride-time, walking speed) parameters can be derived. More in-depth analysis utilising temporal-spatial parameters and participant metadata can describe parameters indicating motor control [38]. Non-linear or phase plot analysis can be used to explore subtle gait changes using the whole dataset of a particular participant. Using this approach, it has been demonstrated that changes in gait can be detected in the pre-symptomatic phase of Huntington’s disease [39].

Although parkinsonian features are generally considered to be later clinical manifestations in AD, it has been reported that parkinsonian features may precede the onset of frank dementia [40, 41]. A standardised neurological examination includes the MDS-Unified Parkinson’s Disease Rating Scale (UPDRS) Part III (Motor) [42], which quantifies presence of tremor, bradykinesia, rigidity, postural instabilty and gait disorder. Assessments are video-taped for quality control purposes and to enable futher review by a senior neurologist if clinically-significant parkinsonian features are identified. The Bradykinesia Akinesia Incoordination (BRAIN) test is administered to all participants via a laptop (Lenovo Thinkpad, Lenovo Group Ltd). This computer keyboard-tapping task was originally developed for use in assessing the effect of symptomatic treatment on motor function in Parkinson’s disease. An online version has been designed and validated which can be utilised as an objective longitudinal measure of emerging motor dysfunction [43]. The outputs of the BRAIN test include a kinesia score (KS30, number of key taps in 30 s), akinesia time (AT30, mean dwell time on each key in ms), incoordination score (IS30, variance of travelling time between key presses) and dysmetria score (DS30, accuracy of key presses).

#### Cognitive battery

The cognitive assessment battery is based on a review of results and cognitive protocols from several large-scale initiatives and clinical trials involving individuals at risk for AD [10, 12, 44], and is complementary to cognitive assessments performed as part of the most recent (in some cases concurrent) NSHD home visit (that includes the ACE-III [45] and word-list learning [46]). Complementing ongoing work with the whole NSHD cohort, participants’ cognitive trajectories will be assessed prospectively over the two time points, and retrospectively using previously collected cognitive measures (refer to Appendix 2 for an overview of neuropsychometric tests collected to date).

The battery includes:

*The mini-mental state examination (MMSE*) [47]

The MMSE is a widely used 30-point screening tool for cognitive impairment within clinical practice, assessing multiple cognitive domains including: *i)* orientation to time and place (10 points); *ii)* registration (3 points); *iii)* attention +/− calculation (5 points); *iv)* recall (3 points); *v)* language (2 points); *vi)* repetition (1 point); *vii)* reading (1 point); *viii)* writing (1 point); *ix)* visuospatial function (1 point); *x)* following a 3-stage command (3 points).

*Logical memory from the Wechsler Memory Scale-Revised (WMS-R*) [48]

The Logical Memory test assesses free recall of a short story that contains 25 details. The participant is asked to recall the story immediately and after a delay of approximately 20 min.

*Digit-symbol substitution test, from the Wechsler Adult Intelligence Scale-Revised (WAIS-R)* [49]

The Digit-Symbol Substitution test explores attention and psychomotor speed. Participants are given a code table displaying the digits from 1 to 9, each paired with a symbol. On a worksheet printed with rows of digits, participants are asked to fill in the corresponding symbol under each digit as shown in the code table, as quickly and accurately as possible. The score is the number of symbols completed correctly within 90 s.

*Matrix reasoning from the Wechsler Abbreviated Scale of Intelligence (WASI*) [50]

The Matrix Reasoning test assesses non-verbal reasoning. Participants are shown a matrix of geometric shapes with a section missing and are required to select the missing piece from five options. There are 32 matrices, graded in difficulty, and the test is discontinued when participants reach a certain error threshold, as specified in the WASI manual.

**Fig. 2:**
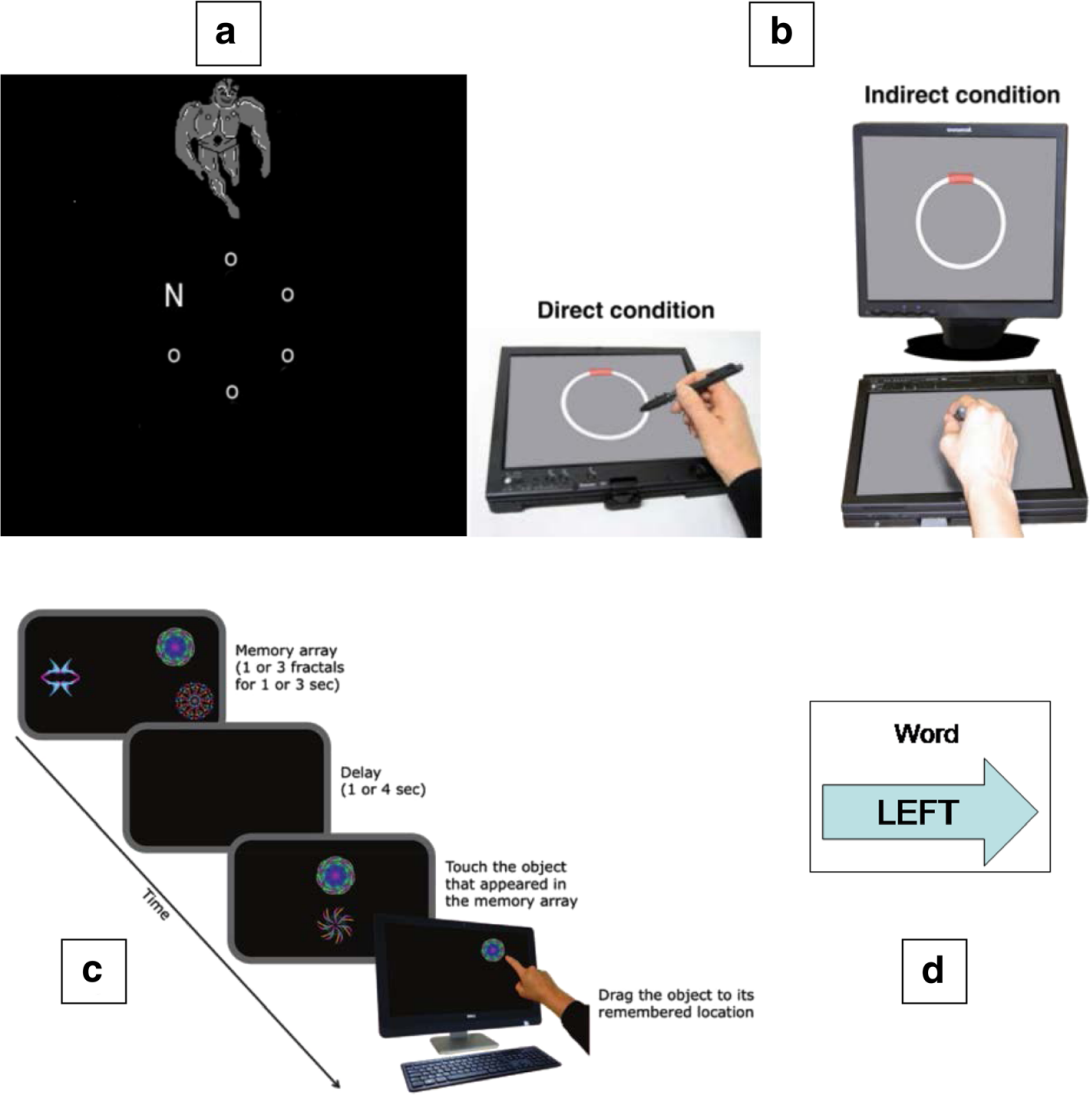
Novel computerised tests (**a**) Irrelevant Distractor. An example stimulus display (not to scale) with an irrelevant distractor in the low load condition. Note that the specific cartoon image shown here as an irrelevant distractor is included for illustrative purposes only, in order to avoid violating copyright for the images used in the experiment. Figure reprinted from [64] with permission from American Psychological Association (**b**) Visuomotor Integration apparatus. Note that in the indirect condition, the participant’s hand is covered by a box, not shown here. Figure reprinted from [61], Copyright, with permission from Elsevier. (**c**) ‘What was where?’ task. Figure reprinted from [56] available from https://www.ncbi.nlm.nih.gov/pmc/articles/PMC4360752/, American Psychological Association, copyright under the Creative Commons Attribution License https://creativecommons.org/licenses/by/3.0/ (**d**) Task-set Switching / Response Inhibition. An example stimulus display for an incongruent word trial

Five more novel tests, intended to detect subtle, early cognitive deficits, are also being administered (see Fig. 2).

*Task-set switching / response inhibition* [51, 52]

A meta-analysis of relationships between amyloid burden and cognition in cognitively-normal older adults found evidence of an association between amyloid burden and executive functioning [53]. This task examines the relationship between two important executive functions – task-set switching and response inhibition – which are vulnerable in early AD [54, 55]. Individuals are presented with a computer screen on which a stimulus is displayed, and a response box with two buttons. The first part of the experiment comprises the simple choice “arrow only” and “word only” conditions, which complements the simple choice reaction time tasks administered at 60–64 years. In the “arrow only” condition, participants are shown the cue ‘arrow’ for 1000 ms, followed by an arrow pointing left or right. In the “word only” condition, participants are shown the cue ‘word’ for 1000 ms, followed by the word ‘left’ or ‘right’. In each case they must press the button that corresponds to the stimulus, using the index and middle fingers of their dominant hand. The second part of the experiment is a switching condition in which the cue may be either ‘arrow’ or ‘word’, and the stimulus is a combined arrow and word. The stimulus is either congruent (e.g. left arrow and the word ‘left’) or incongruent (e.g. left arrow and and the word ‘right’). Trials in the switching task are categorised into switch and non-switch. In a non-switch trial the cue is the same as for the immediately preceding trial, whereas in a switch trial the cue differs from the immediately preceding trial. In order to evaluate the effect of preparation time on task-set switching and response inhibition, the cue is shown for either a short (200 ms) or long (1500 ms) interval before the stimulus appears. Outcome measures are reaction time and error rate.

*‘What was where?’ visual short-term memory binding task* [56, 57]

This test requires participants to view one or three fractal objects, presented simultaneously in random locations on the screen. Participants are asked to remember both the objects and their locations. After a delay of one or four seconds they are required to make a forced choice between two fractals, one of which was displayed in the initial memory array (the target) and the other of which is a ‘dummy’ fractal. Participants are required to touch the object they think has been previously presented and ‘drag’ it on the touch screen to its remembered, original location. Outcome measures are the proportion of fractals correctly identified, and the localisation error (i.e. the distance between the location reported by the participant and the true location of the target in the initial memory array) and the proportion of ‘binding errors’. A binding error occurs when a participant chooses the correct fractal but drags it to the location of one of the non-target (unprobed) fractals from the initial array. The binding of such featural information has been shown to be vulnerable in asymptomatic familial AD mutation carriers [58, 59]. There is evidence that binding ability is relatively preserved in normal ageing despite the age-related decline in memory for object identification and localisation, making it a promising target for sensitive tests to detect preclinical AD [60].

*Visuomotor integration*

This is a circle-tracing task which includes both direct and indirect visual feedback conditions. The task is presented on a tablet laptop, with the screen placed flat on the table in front of the participant, with an additional free-standing monitor behind it. Participants are asked to use a stylus to trace round a circle on the tablet as quickly and accurately as possible. In the direct condition, participants can see their hand and the path they are tracing on the tablet. In the indirect condition, participants put their arm under a box so they cannot see their hand, but they are instructed to look at the free-standing monitor to view a copy of the circle and their tracing path. Continuous performance measures are provided including accuracy, speed and speed of error detection and correction. The test has revealed changes in speed and accuracy in Huntington’s Disease mutation carriers more than 10 years before expected age-of-onset [61].

*12-item Face-Name Associative Memory Exam (FNAME-12A)*

The FNAME-12A is a modified version of the 16-item Face-Name Associative Memory Exam (FNAME-16). The FNAME-12A has fewer stimuli and additional learning trials which are well tolerated by those with mild cognitive impairment (MCI), while remaining challenging in cognitively-normal older adults [62]. It has demonstrated psychometric equivalence with the FNAME-16, which is related to β-amyloid burden in cognitively-normal older people [63]. The FNAME-12A requires the participant to learn 12 face-name and face-occupation pairs. Participants are given two exposures to all 12 face-name/occupation pairs. After each exposure and following a 10-min delay they are asked for the name and occupation associated with each face. After a 35-min delay they are shown three faces and asked to identify each previously learned face from two distractors (facial recognition) and to recall the name and occupation. If they cannot remember the name or occupation, they are provided with three recognition choices.

*Irrelevant distractor paradigm* [64, 65]

Participants are given a computerised letter-search task and are required to make a rapid decision as to which target letter (‘X’ or ‘N’) has appeared in the search display. There are three load conditions, high (four letters), medium (three letters) and low (one letter). On some of the trials, a distractor appears on the outside of the search display. This distractor can either be task-irrelevant (a cartoon character) or task-relevant (the letter X or N). The task-relevant distractors can be congruent or incongruent to the target letter. Outcome measures are reaction time and error rate. The task evaluates the extent to which attention is captured by the different distractors, and the role of perceptual load in this process.

These tasks give weight both to response accuracy and latency to maximize detection of subtle cognitive change and discriminate cortical/subcortical dysfunction. Cognitive performance at the 2-year follow up in the sub-study will be evaluated against study baseline performance and cognitive performance in childhood, adolescence and adulthood.

#### Sensory function

There is increasing interest in the possibility that impaired visual function, sense of smell and hearing may provide signals of preclinical AD [66–70]. As such, participants will have comprehensive sensory assessments of vision, olfaction and both peripheral and central auditory function.

Basic parameters of visual function, an important factor contributing to deficits experienced in elderly patients with cognitive impairment, are assessed using The Portable Eye Examination Kit (PEEK), a smartphone application that measures visual acuity, colour vision and contrast sensitivity [71].

The University of Pennsylvania Smell Identification Test (UPSIT) is a commercially-available, well-established, reliable, and standardized olfactory test that can be self-administered [72]. The ‘British’ version is being used as it is the most culturally appropriate to the NSHD. Each test comprises four 10-page booklets with one odorant (embedded in 10–50-μm diameter microcapsules fixed in a proprietary binder and positioned on brown strips) at the bottom of each page. Accompanying each strip is a multiple-choice question with four responses following an alternative forced-choice paradigm. Packs are provided to participants to complete at home and returned using a pre-paid envelope provided. Participants are also asked prior to testing whether they have subjectively noticed a decline in their sense of smell.

Peripheral hearing is assessed using air conduction threshold audiometry, with pure tones presented to each ear at different frequencies covering the range of human speech perception (0.5 kHz - 4 kHz). A testing procedure in keeping with British Society of Audiology recommendations [73] is used in which the sound level of the tones are varied and enable hearing thresholds for each ear at each frequency to be obtained.

Central auditory processing is tested by using a word identification in background noise paradigm [74]. This involves presentation of high-frequency monosyllabic words embedded in a multi-talker babble noise composed of 20 voices. The background noise is presented at a fixed level of 65 dB SPL, while the sound level of each individual word is varied according to an adaptive staircase procedure based on whether participants are able to identify the word correctly. This is designed to obtain a “speech reception threshold”, which quantifies participants’ ability to identify spoken words in background noise.

### Imaging protocol description

Imaging is performed on a Biograph mMR 3 T PET/MRI scanner (Siemens Healthcare, Erlangen), allowing for simultaneous acquisition of dynamic amyloid PET and MR data whilst minimising scanning time and exposure to radiation (compared with the use of PET-CT). Participants will have one scanning session at each time point. The neuroimaging protocol comprises both structural and functional acquisitions, and is designed to be completed within a 60-min scanning session.

**Fig. 3:**
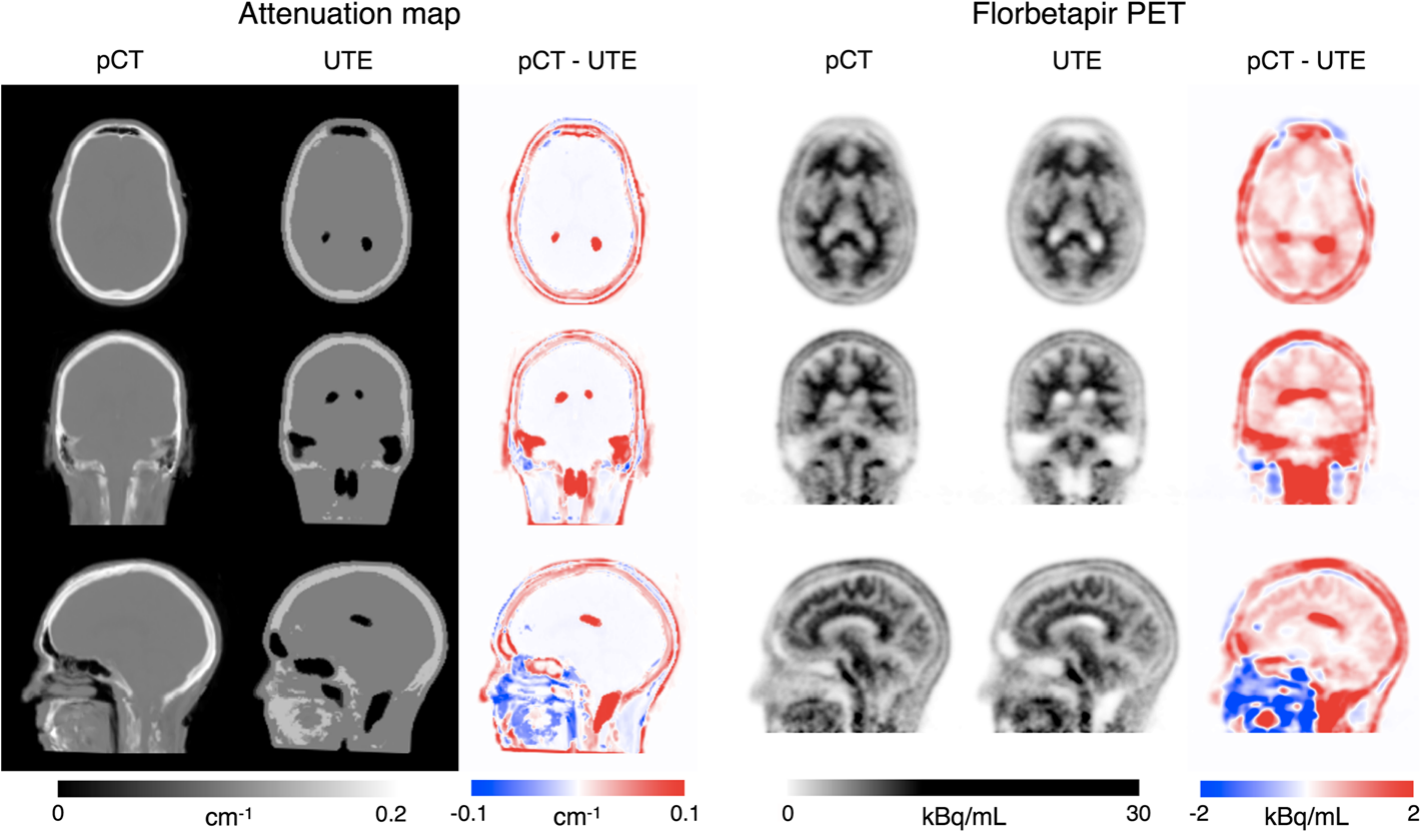
Improved PET reconstruction using the pCT method. Examples of attenuation maps obtained with the multi-atlas CT synthesis method (pCT) and the UTE method and the corresponding florbetapir PET images generated with each method (10-min frame 50 min post-injection). Difference maps are also shown (pCT – UTE) to better visualise the improved PET reconstruction accuracy

Amyloid load is assessed using the ^18^F amyloid PET ligand, florbetapir. Amyloid positivity on florbetapir-PET imaging is correlated with post-mortem Aβ burden, neuritic amyloid plaque density, and neuropathological diagnosis of AD [75]. After intravenous cannulation, 370 MBq florbetapir F18 (Amyvid) is injected. PET data are acquired continuously during and following injection to allow florbetapir uptake dynamics to be assessed. Final amyloid burden is assessed over a 10-min period, ~50 min after injection, with scope for the previous 10-min period to be used if longer scan periods are not tolerated. PET data, acquired in list-mode, is reconstructed using a 3D ordered-subset expectation-maximisation algorithm with three iterations and 21 subsets, and smoothed with a 4 mm Gaussian kernel. Attenuation maps are computed by default from the ultra-short echo time (UTE) sequences provided by the vendor as well as from the T1-weighted and T2-weighted volumetric scans using a multi-atlas CT synthesis method [76], also known as pseudo-CT (pCT). The latter approach significantly improves PET reconstruction accuracy when compared to the UTE-based correction [77], as demonstrated in Fig. 3.

For the MR acquisitions, a body coil RF transmitter is used in conjunction with a 12-channel receiver array head coil. The maximum gradient strength is 45mT/m along each direction. The MR sequences are:

(i) high resolution 3D T1-weighted, T2-weighted and FLAIR volumetric scans;
(ii) resting state functional MRI (rs-fMRI);
(iii) multi-shell high angular resolution diffusion-weighted MRI (DW MRI);
(iv) a multi-echo 3D gradient echo sequence for simultaneous T2*-weighted/susceptibility-weighted imaging (SWI), quantitative susceptibility mapping and b_0_ field mapping; and.
(v) arterial spin labeling (ASL) for quantitative mapping of cerebral blood flow (CBF).

An additional b_0_ field map is also acquired for distortion correction of the rs-fMRI and DW MRI images. Full details of the acquisition parameters are shown in Table 4.

**Table 4:** MRI sequence parameters

	MPRAGE (3D T1)	SPACE (3D T2)	IR-SPACE (3D FLAIR)	rs-fMRI	3D T2*/SWI	Diffusion	Field mapping	ASL
Voxel resolution (mm^3^)	1.1 × 1.1 × 1.1	1.1 × 1.1 × 1.1	1.1 × 1.1 × 1.1	3 × 3 × 4	0.86 × 0.86 × 1.5	2.5 × 2.5 × 2.5	3 × 3 × 3	3.75 × 3.75 × 4
Matrix size	256 × 256 × 208	256 × 256 × 176	256 × 256 × 176	64 × 64 × 36	256 × 192 × 96	96 × 96 × 58	64 × 64 × 55	64 × 56 × 36
FoV (read x PE) (mm)	282 × 282	282 × 282	282 × 282	192 × 192	220 × 165	240 × 240	192 × 192	240 × 210
Slice coverage (mm)	229	194	194	144	144	145	165	144
Orientation	Sagittal	Sagittal	Sagittal	Transverse obl	Transverse obl	Transverse obl	Transverse obl	Transverse obl
PE direction	A > > P	A > > P	A > > P	A > > P	R > > L	A > > P	R > > L	A > > P
TE (ms)	2.92	409	402	30	4.92; 9.84; 19.2	103	4.92; 7.38	20.26
TR (ms)	2000	3200	5000	2020	27	8000	688	4000
Flip angle (°)	8	Variable	Variable	75	15	90/180/180	60	90/160/160/160...
Acq bandwidth (Hz/pix)	240	751	751	2112	400/400/140	1578	260	2298
Parallel imaging	×2	×2	×2	×2	×2	×2	None	None
Total scan time	5 min 06 s	4 min 43 s	6 min 27 s	9 min 27 s	3 min 48 s	10 min 16 s; 5 min 28 s	1 min 31 s	5 min 20s
Other sequence-specific parameters	Water selective excitation pulse TI = 870 ms	Water selective excitation pulse Turbo factor 141 Slice TF 2	Water selective excitation pulse Turbo factor 141 Slice TF 2 T2 sel IR TI = 1800 ms	Fat saturation 277 volumes	$$ \raisebox{1ex}{$7$}\!\left/ \!\raisebox{-1ex}{$8$}\right. $$ partial Fourier along PE1/2 Flow comp for first TE Monopolar read gradient	2 non-zero b- values: b = 2000 s/mm^2^ 64 dir b = 700 s/mm^2^ 32 dir 12 x b = 0 s/mm^2^ interspersed ¾ partial Fourier	2D multi-slice dual gradient echo	10 averages pCASL labeling 4-shot 3D GRASE Turbo Factor 14 EPI Factor 28 Labeling duration 1.8 s Post-labeling delay 1.8 s Background suppression

#### 3D volumetric scans (T1, T2 and FLAIR)

Three volumetric scans are acquired with matched spatial coverage, resolution and complementary contrasts, to aid tissue segmentation, delineation of the intracranial vault, and white-matter lesion visualization. 3D T1-weighted images are obtained using an MPRAGE sequence [78]. This is optimized to provide strong contrast between white matter and grey matter and enable quantification of grey matter macroscopic structures in both cortical and subcortical brain regions. 3D T2-weighted images use a long echo train turbo spin echo sequence (SPACE) [79]. FLAIR images are acquired using the same SPACE sequence as T2-weighted images but with the addition of an inversion preparation pulse to null signal from cerebrospinal fluid. T2-weighted and FLAIR images are sensitive to white-matter lesions and hyperintensities, which are typically seen in association with cerebrovascular disease.

Images undergo manual QC in line with protocols developed for commercial trials, by a trained team who assess motion, coverage and other issues. T1 scans are additionally checked specifically for blurring, image wrap-around and contrast problems, and FLAIR for good CSF suppression.

**Fig. 4:**
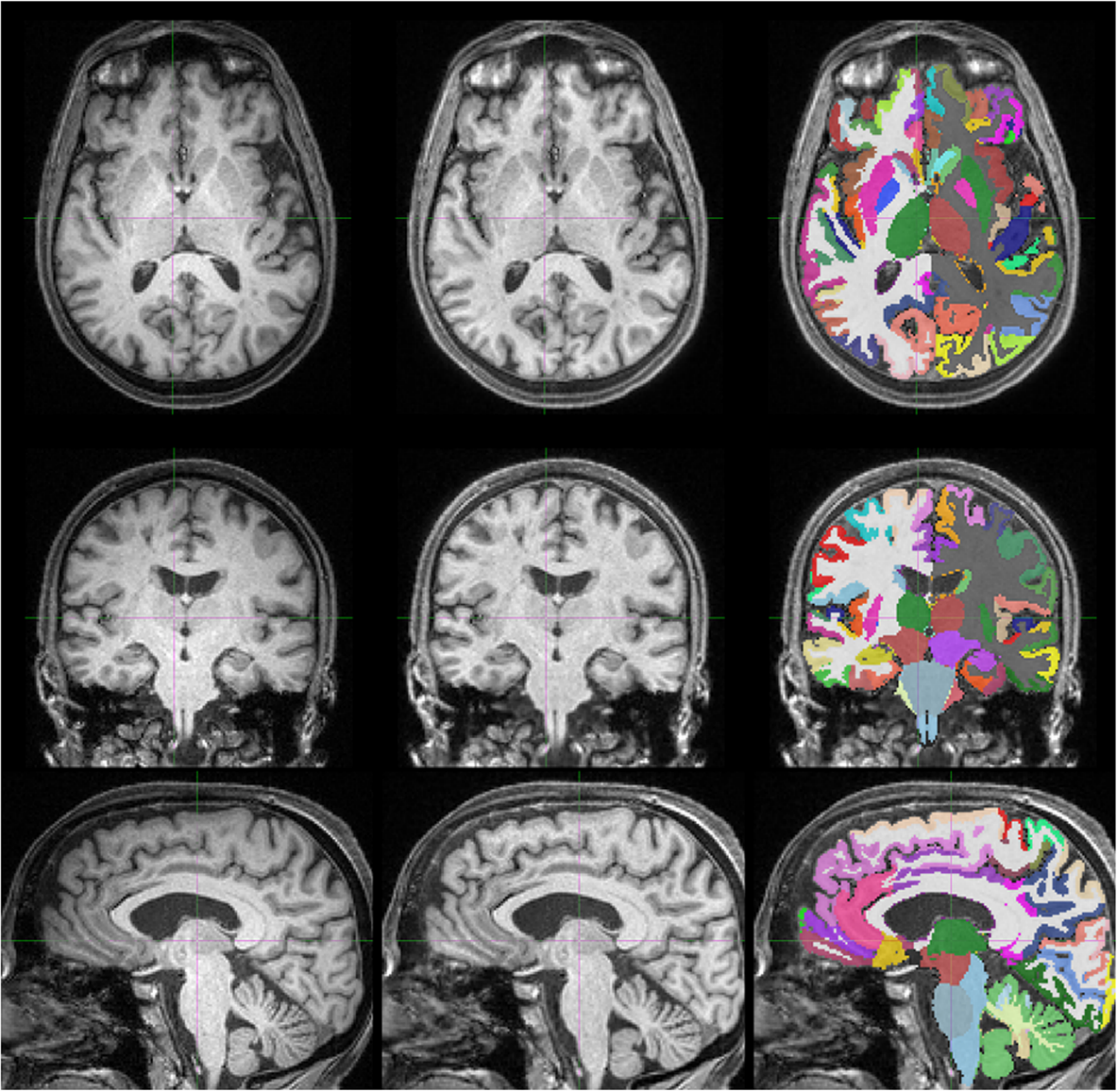
Volumetric T1 pre-processing and segmentation in Insight 46. Examples of axial (*top row*), coronal (*middle row*) and sagittal (*bottom row*) slices from an original MPRAGE volumetric T1 scan (*left column*), pre-processed T1 (distortion and bias field corrected) (*middle column*), and with the GIF parcellation overlaid on top (*right column*)

Pre-processing of structural (T1, T2, FLAIR) images is carried out by applying a correction for gradient non-linearity [80] followed by brain-masked (by registration of MNI template to the scan) N4-bias correction [81]. An automated multi-region parcellation of the T1 images is carried out using geodesic information flow (GIF) [82] – demonstrated in Fig. 4. The parcellation is transferred to microstructure, PET, ASL and fMRI maps for the purpose of region-of-interest (ROI)-based analysis following registration of those images to the T1 image.

#### Resting state fMRI (rs-fMRI)

Resting state, or task-free, fMRI (rs-fMRI) allows for assessment of functional cortical connectivity through analysis of resting state networks (i.e. disparate brain regions which exhibit synchronised fluctuations in local cerebral blood flow while the brain is not engaged in any specific task), which are thought to represent cortical networks critical to the functional organisation of the brain [83].

rs-fMRI is acquired using a T2*-weighted gradient echo EPI acquisition scheme. A resolution of 3x3x4mm^3^ was chosen to ensure whole brain coverage (defined as 144 mm coverage in the inferior-superior direction for angled transverse slice orientation [84]) within a reasonable relaxation time (TR) (~2 s). Parallel imaging is also used to accelerate the imaging and reduce image distortions (GRAPPA with acceleration factor 2). At the beginning of the rs-fMRI acquisition, participants are asked to “close their eyes and not to fall asleep” for the duration of the scan.

Individual fMRI time series are visually checked for head coverage, motion, signal dropout and other artefacts. All fMRI volumes are realigned to correct for motion between individual points in the time course. A plot of the registration parameters computed in the motion correction is provided for manual review to ensure that the level of motion is not severe enough to adversely affect the resulting analysis. Signal quality metrics such as signal-to-noise ratio (SNR), variance of signal change from average signal (DVARs) and spike levels are also plotted over the length of the time course [85]. Timepoints that are outliers are automatically removed from the analysis.

The motion-corrected fMRI time course is then registered to the corresponding anatomical T1 image. A cohort specific group template discretised in MNI space is iteratively computed by mapping all T1 images with 10 (1 rigid, 9 affine) and 10 non-linear registrations into the MNI image space [86]. The fMRI scan is then transformed into the template space by combining the affine registration from fMRI to T1 image with the transformation that maps the individual T1 image into the group template in MNI space. A Generalised Linear Model (optimised with restricted maximum likelihood estimation (REML) [87]) is used to account for signal drifts and physiological noise using cosine basis functions (highpass filtering of frequencies >0.01 Hz), the demeaned motion-realignment estimates and their derivatives, and RETROICOR regressors, where appropriate [88].

Two methods of analyzing the pre-processed data will be used: a seed-based method and independent component analysis (ICA) [89]. In brief, the remaining residuals are smoothed (Gaussian smoothing kernel with 5 mm FWHM) and mapped into the subsampled group space to create spatial correspondence among individual brains. A seed region is chosen to extract an average time course that is correlated with the time course of every individual voxel. The resulting correlation map per participant is Fisher z-transformed to enable t-test hypothesis testing among participants. For the ICA, time courses of the motion-realigned fMRI scan within a mask of the brain are extracted, centered and variance-normalized, resulting in one voxel-time matrix per participant. All participant matrices are then concatenated in time. The obtained group matrix is reduced to its principal components and whitened. The independent component analysis [90] is applied to the whitened group matrix to obtain spatial components. The representation of all group-independent components in each participant is required for group comparison. Dual regression will be applied to obtain group-independent component representations in each participant.

#### Diffusion-weighted MRI

Diffusion-weighted MRI is a technique that enables characterisation of the microstructural integrity of white and grey matter. The majority of studies investigating neurodegeneration utilise the diffusion tensor model [91], which aggregates the differential diffusion profiles of water molecules in extra- and intracellular spaces to produce an array of metrics including fractional anisotropy (FA), axial diffusivity (AxD), radial diffusivity (RD) and mean diffusivity (MD). These metrics have been used to demonstrate change in white-matter tract integrity and grey-matter microstructure in AD [92]. However, this model does not account for multiple fiber orientations or tissue compartments within a voxel, making interpretation of changes in these metrics ambiguous. Neurite orientation dispersion and density imaging (NODDI) [93] is a recently-developed multi-shell diffusion technique which allows for the estimation of tissue microstructure at the sub-voxel level by assuming that water protons in neuronal tissue can be considered to be in three different pools: i) free water, modelling CSF space; ii) restricted water, modelling dendrites and axons; and iii) hindered water, modelling diffusion within glial cells, neuronal cell bodies and the extracellular environment. This more complex modelling enables estimation of neurite density (neurite density index (NDI)) and neurite orientation dispersion (orientation dispersion index (ODI)) in both white and grey matter.

Diffusion MRI is acquired using a twice-refocused spin echo EPI sequence [94, 95] with two non-zero b-values (700 and 2000 s/mm^2^) and multiple directions (32 and 64 directions for the b = 700/2000 s/mm^2^ scans respectively). The b-vector directions were calculated to be uniformly distributed over a hemisphere, and images with b = 0 s/mm^2^ are interspersed throughout the acquisition (12 obtained overall). Images are acquired with an isotropic 2.5 × 2.5 × 2.5mm^3^ resolution, with 58 slices to ensure whole brain coverage.

Visual review is performed for identification of poor quality images by checking for: (i) full brain coverage; (ii) inter-acquisition motion (using motion plots over the acquisition); (iii) sufficient correction of geometric distortion; and (iv) slice-wise signal dropout (using correlation plots between adjacent slices). Images failing this quality-control process are removed before running the diffusion analysis. For each participant, if the number of acquisitions that have failed is high enough that it might affect the consistency of the analysis between subjects, then these data are marked as a ‘failed acquisition’.

**Fig. 5:**
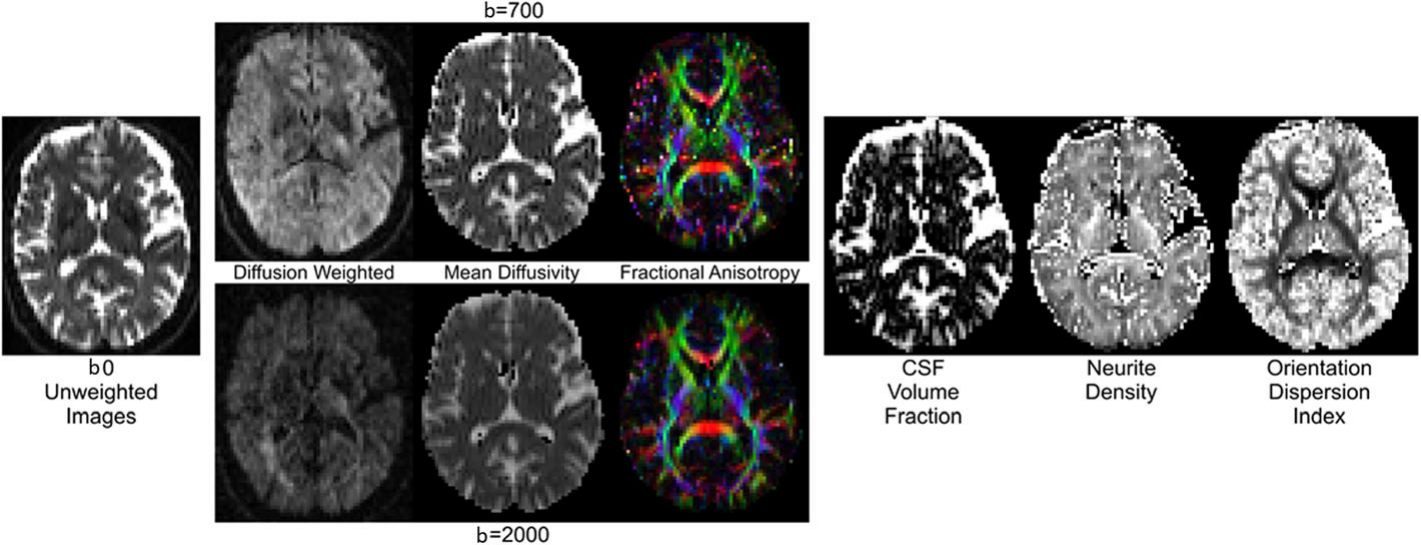
Representative diffusion images in Insight 46. Examples of diffusion images at the two b values, b = 700 and 2000 s/mm^2^, with their corresponding derived MD and FA maps (*left*) and NODDI metrics (*right*)

Pre-processing of diffusion-weighted images involves first correcting for inter-volume motion registration and eddy currents using FSL’s Eddy tool [96]. This is followed by correction for EPI susceptibility distortion using field maps to the structural T1 [97] and phase-encode direction constrained non -linear registration to the T1 volume, with modulation based on the Jacobian determinants. The separate diffusion-weighted shells (together with their associated b = 0 volumes) are fitted with a diffusion tensor model using NiftyFit [98]. The NODDI model is then fitted to the combined shells [93] (see Fig. 5 for an example of the diffusion-weighted images and corresponding parameter maps).

#### 3D T2*/SWI

T2*/SWI are iron-sensitive sequences that allow for detection of cerebral microbleeds, associated with cerebral small vessel disease or cerebral amyloid angiopathy, generally distinguished by their distribution within the brain parenchyma. Microbleeds are more common in AD than controls [99], and may independently impact on cognitive function with potential mechanisms including tissue necrosis in strategic white-matter tracts [100], or via the initiation of an inflammatory cascade [100].

A 3D multi-echo gradient echo sequence is acquired to generate T2*-weighted images, T2* maps, SWI, and quantitative susceptibility maps (qSM). This sequence can also provide b_0_ field mapping at higher resolution than the GRE-based field maps that are currently often used for processing geometric distortion correction of the DWI and fMRI. The sequence acquires magnitude and phase images at three echo times: TE = 4.92, 9.84, and 19.7 ms (chosen to keep fat and water signals in phase). Magnitude images from the longest TE (19.7 ms) provide the T2*-weighted volume, which are combined with the phase images from the same echo time to generate SWI [101]. T2* maps are generated by fitting the three magnitude images to a monoexponential decay S = S0.exp.(−TE/T2*), and qSM are obtained from the three phase images using the superfast dipole inversion (SDI) method [102]. The images are visually checked for coverage, motion, and artefacts.

#### Arterial spin labelling (ASL)

ASL is a non-invasive method for determining regional cerebral blood flow (CBF). In brief, blood flowing through the neck vessels is labelled via spin inversion. After a delay period to allow this labelled blood to perfuse into the brain, images are acquired, and compared with equivalent images acquired without prior spin labelling (unlabelled images). The signal difference between labelled and unlabelled images is proportional to blood flow, thus enabling calculation of a quantitative cerebral blood flow map.

ASL data are acquired using a 4-shot segmented 3D GRASE pCASL sequence [103] (for more details refer to Table 4). The ASL timing parameters were chosen based on the recommendations of the recent ISMRM Perfusion Study Group consensus paper [104]: labeling duration =1800 ms; post labelling delay =1800 ms. Ten averages are acquired and saved separately, in order to allow image realignment prior to averaging. Background suppression is used to reduce static tissue signal, and an accompanying set of three saturation recovery data sets (TR = 1 s, 2 s, /2/4 s) are acquired using the same 3D GRASE acquisition scheme for estimation of tissue T1 and M0 (total scan time ~ 50s).

During pre-processing, CBF maps are calculated using the recommended model for ASL images acquired with a single post-labelling delay [104], implemented in NiftyFit [98] and assuming blood T1 at 3 T of 1650 ms and an overall labelling efficiency of 0.833 (blood inversion efficiency of 0.85 and two background suppression pulses with inversion efficiency 0.99 each). The saturation recovery data are fitted to a monoexponential recovery curve to estimate the underlying tissue magnetisation (M0) and receiver coil sensitivity variation, enabling generation of quantitative CBF maps [98]. Error maps are also provided to allow inference of how precisely CBF in different regions has been estimated.

### Primary imaging pipelines and analyses

All imaging data, derived results, visual quality checks, and radiological reads are stored on a customised web-based server running XNAT 1.6.5 (www.xnat.org). As protected health information (PHI) is stored in the proprietary listmode format, but difficult to remove, the decision was made not to enter any PHI on the scanner console of these participants. After data are acquired on the PET/MR scanner they are transferred to a study-specific waypoint. This includes both the DICOM-compliant imaging format used for MRI and PET images that are reconstructed on the scanner and raw listmode data that consists of a customised DICOM file which contains the Interfile header and a corresponding binary data file generated from the full 60 min of PET acquisition. Twice daily, all recent data are securely synchronised between the study-specific waypoint and the XNAT server, where separate import processes are done for both the DICOM and listmode data. DICOM data are checked for completeness before formally importing the data into the XNAT database. Once the DICOM data have been imported, listmode data are then added to the PET-MR imaging session in the database and automatic checks are performed to ensure that they contain the full 60-min acquisition, including some small amount of time before the tracer was injected. Next, an in-house workflow management system automatically starts the modality-specific pre-processing steps mentioned in the previous section. Key derived images generated during these pre-processing steps are stored on the XNAT server by attaching them to the original imaging session. Once pre-processing is complete, visual review for each modality is performed and a customised modality-specific webform of the quality checking is stored on the XNAT server.

A consultant neuroradiologist reviews all T1, T2 and FLAIR sequences as outlined in the “Duty of Care” section by downloading the key imaging data from the server and completing a customised radiological read web-form within XNAT. Customised reports are provided to the neuroradiologist which identify the PET-MR imaging sessions where a read needs to be completed.

**Fig. 6:**
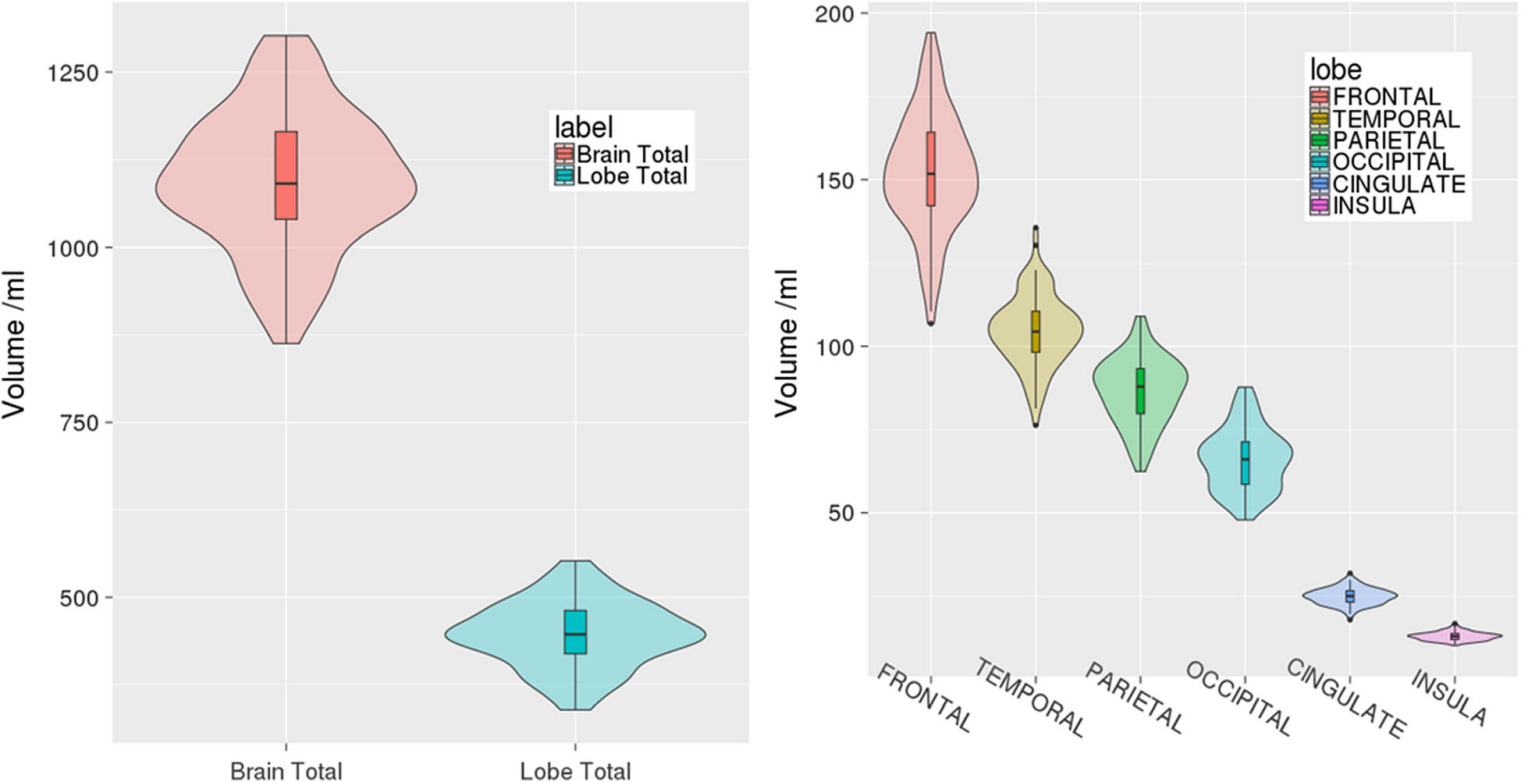
Brain volumes derived from first 100 Insight 46 volumetric T1 scans. Violin plots demonstrating total brain and lobar volumes (*left*) and regional lobar volumes (*right*) calculated on the first 100 T1 scans in Insight 46 using the automated segmentation pipeline

Primary analyses of T1 images include automated segmentation of whole-brain [105] and hippocampal regions [106], followed by manual checking and editing, semi-automated ventricle segmentation, automated total incracranial volume (TIV) measurement [107] and semi-automated cortical thickness calculation [108]. Figure 4 provides an example of the GIF segmentation and Fig. 6 shows summary volumetric metrics from the first 100 T1 scans. White-matter burden and micro haemorrhages are assessed using visual rating scales [109, 110], and automated quantification [111, 112].

Volume loss between serial scans will be determined using the robust boundary shift integral (BSI) [113] following affine registration of repeat and baseline scans. Regional BSI calculation is performed using a fully affine whole-brain registration followed by rigid registration of masked local structures. Tensor-based morphometry is used as a non-region-based measure of volume loss.

The amyloid PET images are registered to the T1-scan and visually rated as positive/negative by experienced nuclear medicine specialists [114]. Standardised uptake value ratios (SUVR) are calculated globally and locally from a T1-parcellation [115]. Regional grey matter amyloid load is calculated with appropriate partial volume correction [116].

### Blood and urine specimen protocol for clinical samples and biomarker identification

Blood samples are collected for haemoglobin, platelet count, urea and creatinine, random glucose, vitamin B12, and TSH, as potential modulators both of cognition and progression of Alzheimer’s pathology. Samples will also be stored for biomarker exploration (both serum and plasma) and genetic analysis. Blood is collected using a Vacutainer system in a single venepuncture procedure. Samples are inverted eight times to ensure adequate mixing of blood with tube additives. All samples are processed within two hours of collection, as per recent working group guidelines on biomarker pre-processing [117]. One 4.0 ml EDTA sample is collected for haemoglobin and platelet count and the remaining sample is stored at -20 °C prior to genetic extraction. One 2.5 ml SST sample is collected for urea, creatinine, TSH, vitamin B12 and random glucose. Two 10 ml EDTA samples and two clotted 8.5 ml SST samples are spun at 2000 g for 10 min to generate up to 8.0 ml plasma stored as 16 × 0.5 ml aliquots and up to 7.0 ml serum stored as 14 × 0.5 ml aliquots, all at -80 °C, for later analysis. Aliqot tubes are made from polypropylene. Urine is collected in a 100 ml polypropylene pot and transferred on ice for storage. A total of 24 ml urine is stored across 5 aliquots at -80 °C. Planned analyses include measurement of serum neurofilament light [118], plasma tau [119] and plasma Aβ40 and Aβ42 [120] using ultrasensitive Single molecule array (Simoa) assays [121].

### Genetics

DNA from each participant is extracted from whole blood using standard methods (phenol-chloroform). Existing genotyping data are available from the Metabochip [122] and the DrugDev Consortium Array (Illumina, inc.) and samples are further assayed on NeuroX2 (Infinium NeuroConsortium Array, Illumina, inc.) according to the recommended protocol. This genotyping platform is the second iteration of a neurological disease-specific array. It covers approximately 500,000 genetic markers, many of which will have a role in neurodegenerative disease, and improves on NeuroX which was designed and released in 2014 [123]. NeuroX2 includes an up-to-date content, with the latest signals from the most recent genome-wide association study (GWAS) in neurodegenerative diseases, such as PD and AD. As an example, all known genome-wide associated and suggestive loci for AD are present in the array, which enables the creation and ascertainment of polygenic risk scores for that disease [15], which can then be improved by using biomarker and imaging data from the same participants.

### Analysis plan

Overarching themes of the study include better characterisation of the prevalence and incidence of cerebral amyloidosis in a British birth cohort; examining how biological, genetic, psychological and social factors across life influence cerebrovascular burden, amyloid burden, and neurodegeneration (as measured by cortical network breakdown and cerebral atrophy), and their interplay on cognition; and in doing so establishing metrics that are sensitive for detecting early neurodegeneration.

Specific research questions we will address include: the extent and variability of amyloid deposition and proportion of this representative cohort who will be amyloid positive (estimated at 15–25%); the relationships between amyloid load, standard and more advanced MR metrics and vascular burden; the influence of childhood cognitive and motor ability, educational attainment, lifetime mental health, physical activity, physical function and cerebrovascular risk profiles and genetic factors on the development of brain pathology, cognitive health, gait and motor skills; the cognitive tests – both established and novel – that are most associated with cross-sectional biomarkers of brain pathology and have most power to detect change over time and thus to be outcome measures for clinical trials; the extent to which genetic risk scores and blood-based biomarkers can detect asymptomatic amyloidosis; and how best to recruit to presymptomatic AD trials and which imaging and other biomarkers will maximise power to detect treatment effects in the preclinical and very early stages of cognitive decline.

## Discussion

Insight 46 intends to integrate the NSHD data on the socioeconomic environment and on physical, psychological and cognitive function from 0 to 69 years, coupled with data on genetics, structural and molecular imaging, and intensive cognitive and neurological phenotyping, to inform what influences the entire spectrum of changes that occur as the brain ages: from healthy through to pathological ageing, with a specific focus on AD. Combining the cohort’s uniquely rich life course data with the metrics collected in Insight 46, at an age when overt dementia is rare, provides an unprecedented opportunity to explore pre-symptomatic neurodegeneration and to evaluate very subtle cognitive decline. Prospective follow up in the sub-study allows for the consequences of these changes to be quantified. These analyses, initially performed in house, with subsequent data dissemination in line with the MRC Policy on data sharing, have the potential to provide fundamental insights into the factors that influence healthy and pathological brain ageing, provide an evidence base to inform how best to identify individuals at high risk for AD and other forms of dementia, and contribute to practices for monitoring change over time for clinical trials.

## Appendix 1

**Table 5:** Summary of reportable findings and normal ranges used in Insight 46

Measurements with Reportable Results	Normal range/Non-reportable Findings	Reportable Findings and Associated Duty-of-care Actions
Blood pressure	Systolic 91–139 mmHg Diastolic 61–84 mmHg	Severely low AND symptomatic: Systolic ≤90 or diastolic ≤60 mmHg — recommended to visit GP within five days Mildly raised: Systolic 140–159 or diastolic 85–99 mmHg — recommended to visit GP within three months Moderately raised: Systolic 160–179 or diastolic 100-114 mmHg — recommended to visit GP within 2–3 weeks Severely raised: systolic ≥180 or diastolic ≥115 mmHg — recommended to visit GP within five days
Blood results	• Glucose 3.5–10 mmol/l • Haemoglobin Female 11.5–15.5 g/dl Male 13.0–17.0 g/dl • Platelets 150–400 10^9^/l • Urea 1.7–8.3 mmol/l • Creatinine Female 49–92 μmol/l Male 66–112 μmol/l • Vitamin B12 191–900 pg/ml • TSH 0.27–5.5 mIU/l	Out of normal range: The study member is advised to see their GP within an appropriate time frame dependent on the result and the GP is informed. Action level: Results are discussed with the study member during a post-visit telephone call and are advised to see their GP urgently. The study member’s GP is contacted and receives results within 48 h of clinic visit. The action level for blood results are as follows: • Glucose >20 mmol/l • Haemoglobin <10 or >20 g/dl • Platelets <100 or >1000 10^9^/l • Urea >20 mmol/l • Creatinine >200 μmol/l • Vitamin B12 < 100 pg/ml • TSH <0.1 or >10 mIU/l
Pure Tone Audiometry	Thresholds <35 dB in the range of 0.5–4 kHz	Results are available on the day of the visit and reported to the GP. If any of the thresholds in either ear are ≥35 dB or if there is a difference between the ears of ≥20 dB at two or more frequencies in the range 0.5–4 kHz, the participant is advised to consult their GP.
Mini-Mental State Examination (MMSE)	25–30	A score ≤ 24 results in a letter asking the GP to consider the findings in the context of the study member’s known background (education, medication history, anxiety, depression, etc). The study member receives a letter suggesting they make an appointment to see their GP.
Movement Disorder Society Unified Parkinson’s Disease Rating Scale (MDS-UPDRS)	Queen Square Brain Bank criteria for parkinsonism not met	Only clear and previously undiagnosed Parkinson’s disease will be reported to study members and their GPs. This will be based on the video and clinical assessment (including UPDRS score) performed by a clinical research associate. Where the individual is found to fulfil Queen Square Brain Bank criteria for parkinsonism, a letter will be sent to the GP explaining the findings and recommendations for clinical action. A letter will also be sent to the study member stating that an abnormality has been found and advising them to contact their GP. *Note:* Queen Square Brain Bank criteria for parkinsonism: • Bradykinesia and • At least one of the following: o Muscular rigidity; o Rest tremor; o Postural instability.
MRI	The following incidental findings will not be routinely fed-back to individuals or their GPs: • White matter hyperintensities; • Suspected demyelination; • Non-acute brain infarction; • Chronic hydrocephalus; • Asymmetric ventricles; • Lipoma of corpus callosum; • Developmental abnormalities (including venous anomalies); • Enlarged cisterna magna; • Enlarged perivascular spaces; • Chiari malformation; and • Hippocampal or other focal brain atrophy.	Urgent events during/around scanning: Any emergency occurring around the time of the scan will be dealt with by the clinical research associate, and the participant will be sent to A&E. Non-urgent incidental findings: The following incidental findings will be flagged as reportable by a consultant neuroradiologist and will be reported to participants and their GPs: • Acute brain infarction; • Acute brain haemorrhage (note: not old bleeds); • Intracranial mass lesions (note: not meningiomas in locations considered highly unlikely to cause problems); • Suspected intracranial aneurysm or vascular malformation (including cavernomata) (note: not aneurysms less than 7 mm in diameter); • Colloid cyst of the 3rd ventricle; • Acute hydrocephalus; • Significant sinus disease with suspicion of underlying pathology (e.g., unilateral sinus opacification); and • Other unexpected, serious, or life-threatening findings. Where a reportable incidental finding is identified, the GP will be advised of the nature of the abnormality, what clinical action is recommended. A letter will also be sent to the participant stating that an abnormality has been found and advising the participant to contact his or her GP.

## Appendix 2

**Table 6:** Overview of neuropsychometric tests collected to date in the NSHD

Cognitive domain		Age 8 [124–126]	Age 11 [124–127]	Age 15 [124–126, 128]	Age 18 [126]	Age 43 [129–131]	Age 53 [127–130, 132]	Age 60–64 [46, 124, 132, 133]	Age 69	Ages 69–71 and 71–73 (Insight 46)
Premorbid IQ (estimated)							National Adult Reading Test			
Verbal reasoning and reading comprehension		Reading comprehension test	Verbal abilities test	Verbal section of Alice Heim Test; Watts-Vernon reading comprehension test.		Watts-Vernon reading comprehension test				
Non-verbal reasoning		Picture intelligence test	Non-verbal abilities test	Non-verbal section of Alice Heim Test						WASI Matrix Reasoning
Memory	Short-term Verbal Memory					Word list	Word list	Word list	Word list	WMS-R Logical Memory
	Short-term Visual Memory					Visual memory test				‘What was where?’ task
	Short-term Associative Memory									FNAME-12
	Prospective Memory						Prospective memory test			
Literacy		Word reading test; Vocabulary test	Word reading test; Vocabulary test							
Numeracy			Arithmetic test	Mathematics test						
Executive function	Verbal Fluency						Category fluency		Phonemic fluency and category fluency (as part of the ACE-III exam)	
	Reaction Time/ Task-set switching / Response Inhibition							Simple Reaction Time; Choice Reaction Time		Choice Reaction time (inc. switching and inhibition measures)
Processing speed and attention						Letter Search	Letter Search	Letter Search	Letter Search	WAIS-R Digit Symbol Substitution; Irrelevant Distractor
Visuomotor integration						Timed manual pegboard				Circle Tracing
General / multiple domains					Educational attainment measure	Educational attainment measure			Addenbrookes Cognitive Examination (ACE-III)	Mini Mental State Examination (MMSE)

## Abbreviations

3 T: 3 Tesla (magnetic field strength)
ACE-III: Addenbrooke’s Cognitive Examination 3rd revision
AD: Alzheimer’s disease
ASL: arterial spin labelling
AxD: axial diffusivity
Aβ: beta-amyloid
BRAIN: BRadykinesia Akinesia INcoordination test
CBF: cerebral blood flow
CDR: Clinical Dementia Rating scale
DICOM: Digital Imaging and Communications in Medicine, a standard for handling, storing, printing, and transmitting information in medical imaging
DVAR: variance of signal change from average signal in fMRI
DW MRI: diffusion-weighted MRI
EDTA: ethylenediaminetetraacetic acid
EPI: echo planar imaging
FA: fractional anisotropy
FLAIR: fluid attenuated inversion recovery MRI
fMRI: functional MRI
FNAME-12A: 12-item Face-Name Associative Memory Exam
FNAME-16: 16-item Face-Name Associative Memory Exam
FWHM: full width at half maximum (spatial resolution)
GIF: geodesic information flow, an in-house method of MRI segmentation
GRAPPA: GeneRalized Autocalibrating Partial Parallel Acquisition, an MRI multi-coil parallel imaging technique
GRASE: gradient- and spin-echo MRI
GWAS: genome wide association study
ICA: independent component analysis
MCI: mild cognitive impairment
MD: mean diffusivity
MMSE: mini-mental state examination
MPRAGE: magnetisation prepared rapid gradient echo MRI
MRC: Medical Research Council
MRI: magnetic resonance imaging
NDI: neurite density index
NODDI: neurite orientation dispersion and density index
NRES: National Research Ethics Service
NSHD: National Survey of Health and Development
ODI: orientation dispersion index
pCASL: pseudo-Continuous Arterial Spin Labelling
PD: Parkinson’s disease
PEEK: Portable Eye Examination Kit
PET: positron emission tomography
PHI: protected health information
QC: quality control
RD: radial diffusivity
REC: research ethics committee
REM: rapid eye movement
SCD-Q: Subjective Cognitive Decline Questionnaire
SNR: signal-to-noise ratio
SPACE: Sampling Perfection with Application optimized Contrasts using different flip angle Evolution MRI
SST: serum-separator tube
SUVR: standardised uptake value ratio
T1: MRI spin–lattice or longitudinal relaxation time
T2*: MRI measure of loss of phase coherence among spins oriented at an angle to the static magnetic field due to a combination of magnetic field inhomogeneities and the spin-spin relaxation
T2: MRI spin-spin or transverse relaxation time
TE: MRI echo time
TR: MRI repetition time
TSH: thyroid-stimulating hormone
UCL: University College London
UPDRS: Unified Parkinson’s Disease Rating Scale
UPSIT: University of Pennsylvania Smell Identification Test
UTE: MRI ultrashort TE
WAIS-R: Wechsler Adult Intelligence Scale-Revised
WASI: Wechsler Abbreviated Scale of Intelligence
WMS-R: Wechsler Memory Scale-Revised
XNAT: an open-source imaging informatics software platform
